# Different Techniques of Surgical Left Atrial Appendage Closure and Their Efficacy: A Systematic Review

**DOI:** 10.31083/j.rcm2406184

**Published:** 2023-06-27

**Authors:** Mizar D'Abramo, Silvia Romiti, Sara Saltarocchi, Wael Saade, Flaminia Spunticchia, Noemi Bruno, Mariangela Peruzzi, Fabio Miraldi, Giacomo Frati, Ernesto Greco, Francesco Macrina, Paolo De Orchi, Antonino G. M. Marullo

**Affiliations:** ^1^Department of Clinical, Internal Anesthesiological and Cardiovascular Sciences, Sapienza University of Rome, 00161 Rome, Italy; ^2^Department of Cardiology, Mediterranea Cardiocentro, 80122 Naples, Italy; ^3^Department of Medico-Surgical Sciences and Biotechnologies, Sapienza University of Rome, 04100 Latina, Italy; ^4^Department of Angiocardioneurology, IRCCS NeuroMed, 86077 Pozzilli (IS), Italy

**Keywords:** left atrial appendage closure, LAAC, surgical closure, left atrial appendage, atrial fibrillation

## Abstract

**Background:**

Atrial fibrillation has been identified as an independent risk factor for thromboembolic events. Since 1948 different surgical techniques have described the feasibility and the rationale of left atrial surgical appendage closure. The aim of this systematic review is to evaluate the reported patency rates of different surgical techniques.

**Methods:**

This systematic review was conducted according to preferred reporting items for systematic reviews and meta-analyses (PRISMA) guidelines. Two independent investigators searched the PubMed, Scopus, Web of Science, Cochrane Central Register of Controlled Trials, and OVID® (Wolters Kluwer, Alphen aan den Rijn, Netherlands) to identify relevant studies. Consecutively, a PICO (Population, Intervention, Comparison and Outcomes) strategy assessment of literature was performed to search eventual other relevant studies that may have been ignored.

**Results:**

A total of 42 studies were included in our analysis. The total number of patients who underwent surgical left atrial appendage closure was 5671, and in 61.2% an imaging follow up was performed, mostly with transesophageal echocardiographic evaluation. Success rate for the different techniques was: Clip deployment 98%; Lariat procedure 88%; Surgical amputation 91%; Endocardial suture 74.3%, Epicardial suture 65%; Left atrial appendage closure (LAAC) ligation 60.9%; Stapler technique with excision of left atrial appendage (LAA) 100%; Stapler without excision 70%.

**Conclusions:**

To date, data on surgical left atrial appendage closure are poor and not standardized, even if reported rates are acceptable and comparable to transcatheter procedures. If validated on large-scale non-retrospective and multicentric studies, these promising developments may offer a valuable alternative for patients with atrial fibrillation (AF) and ineligible for oral anticoagulation therapy.

## 1. Introduction

Atrial fibrillation (AF) is the most common cardiac rhythm disorder, with an estimated worldwide prevalence of around 46.3 million of people in 2016 with higher incidence according to age and ethnicity [1]. AF has been identified as an independent risk factor for thromboembolic events and is associated with higher incidence of morbidity and mortality due to ischemic stroke and, accordingly, should not be considered a benign disease [1]. The actual risk of stroke in patients with AF is estimated 5% per year, and this percentage may further increase when other risk factors, such as age, hypertension and left ventricular dysfunction, are associated [1]. The left atrial appendage (LAA) has been suspected and therefore studied as a possible source of thromboembolism as early as 1925 [2]. Originally the LAA has been described as a non-functioning anatomical structure, an embryological remnant and subsequently as the “most lethal human attachment” [3]. Nowadays its physiological activity is well established. Function of the LAA includes modulation of the sympathetic and parasympathetic tone, production of the natriuretic peptide balance, left atrium (LA) pressure and volume overload, and contribution to the diastolic filling of the left ventricle [4, 5]. The latter, however, is severely impaired during AF, especially when the LAA presents all the criteria of the Virchow’s triad (stasis; vascular endothelial injury, due to the overstretching of the atrial muscle fibers with fibroblastic infiltration and subsequent inflammation; blood alteration, related to platelet activation and inflammation) [6, 7]. Therefore, in patients with nonvalvular AF, up to 91% of thrombi develop within the LAA compared with patients with valvular AF, in whom LAA localization is ~57%.

## 2. LAA Anatomy and Physiology

**Fig. 1.**
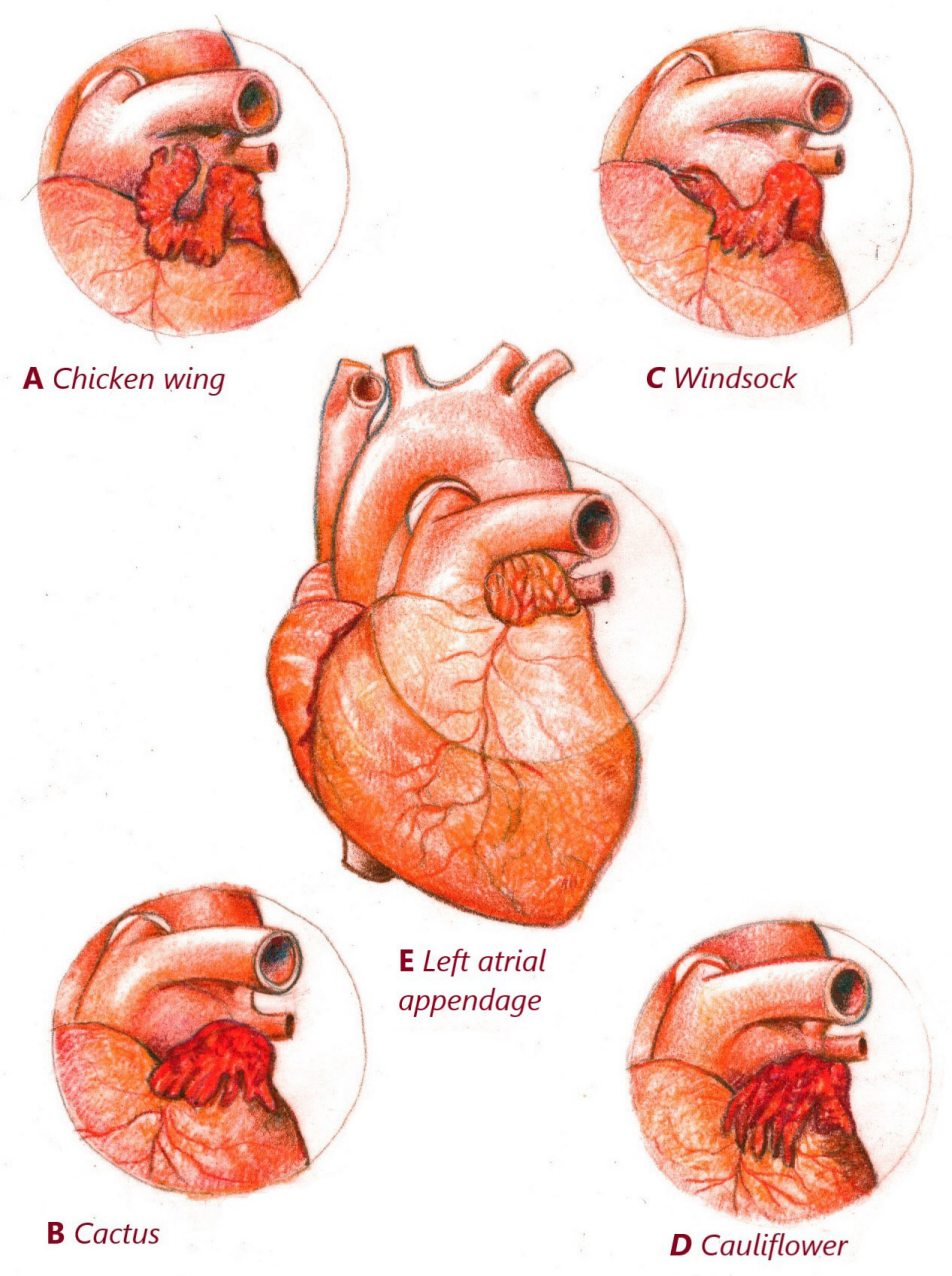
**Left atrial appendage classification according to morphologies**. (A) LAA Chicken wing shaped. (B) LAA Cactus shaped. (C) LAA Windsock shaped. (D) LAA Cauliflower shaped. (E) Left atrial appendage. LAA, left atrial appendage.

LAA can be considered a finger-like extension of the left atrium muscular wall, an embryonic remnant that develops during the fourth week of gestation after the development of the LA that occurs during the third week [8]. On average the LAA has a length of 46 mm and a volume of 9 mL. The LAA lies within the pericardium, anteriorly to the left pulmonary veins and inferiorly to the pulmonary artery, adjacent to the free wall of the left ventricle. Importantly, it’s close to the left phrenic nerve and the left circumflex artery. The LAA can be structurally divided into two parts: the ostium and the body. The ostium represents the point of convergence between the antero-lateral walls of the LA and the LAA pectinate muscles. Several three-dimensional morphologies of left appendage junctions with the LA have been identified on computed tomography: oval-shaped, teardrop-shaped, foot-shaped, triangular, and round-shaped, among which the oval configuration, observed in 68.9% of cases, represents the most common anatomical outline [9]. Moreover, the LAA main body conformation can range from single-lobed, bilobed and, most commonly, trilobed. In a recent classification, based on computed tomography and magnetic resonance imaging, four different LAA shapes were classified: chicken wing (48%), cactus (30%), windsock (19%) and cauliflower (3%) [10] (Fig. 1). According to this classification, chicken-wing morphology is a protective factor in terms of thromboembolic events and is associated with lower thromboembolic risk even in accordance with comorbidities and ${\mathrm{CHA}}_{2}$${\mathrm{DS}}_{2}$ score [10].

The LAA architecture is complex with non-uniform wall thickness consisting of endocardial and epicardial fibers arranged in different orientations [9]. Alterations in LAA flow velocity and structural remodeling of the endothelium are involved in the increased thromboembolic risk in AF patients. In fact, LAA has been shown to possess mechanical and homeostatic properties and a pivotal role in the development of the major AF complications. The LAA flow velocity depends on its morphology, gender, cardiac rhythm, aging, left ventricular function and heart valve disease such as mitral stenosis. Interestingly, chicken wing shaped LAA has been correlated with a higher flow velocity compared with cactus and cauliflower morphologies probably justifying its minor risk of thromboembolic events. In patients with atrial fibrillation LAA flow velocity has been reported to be lower than the one measured in normal sinus rhythm, with an inverse relationship with ventricular rate, age, and female sex [11]. Additionally, the LAA plays a key role in volume homeostasis by producing atrial and brain natriuretic peptides which act increasing renal sodium excretion and, consequently, reducing extracellular volume and blood pressure. Moreover, new evidence suggests an involvement in the regulation of the adrenergic system and renin-aldosterone system (RAA) [12].

## 3. LAA Closure

Considering the pivotal role of LAA in thrombi formation and migration in AF patients, surgical and/or transcatheter LAA exclusion techniques are emerging as safe, feasible and increasingly adopted treatment for mechanical thromboprophylaxis, even in elderly patients [13]. Since 1948, when Madden *et al*. [14] evidenced the feasibility and the rationale of this procedure during mitral valve surgery [15, 16], concomitant surgical closure of the left atrium appendage (sLAAC) in cardiac surgery, even using minimally invasive and video assisted approach [17, 18, 19, 20, 21], was associated with lower risk of cerebrovascular events in patients with AF. Ando *et al*. [22] reported in a systematic review and meta-analysis that sLAAC significantly decreased the risk of mortality and prevented cerebrovascular complication at 30-day follow-up, especially in patients with pre-operative AF.

**Fig. 2.**
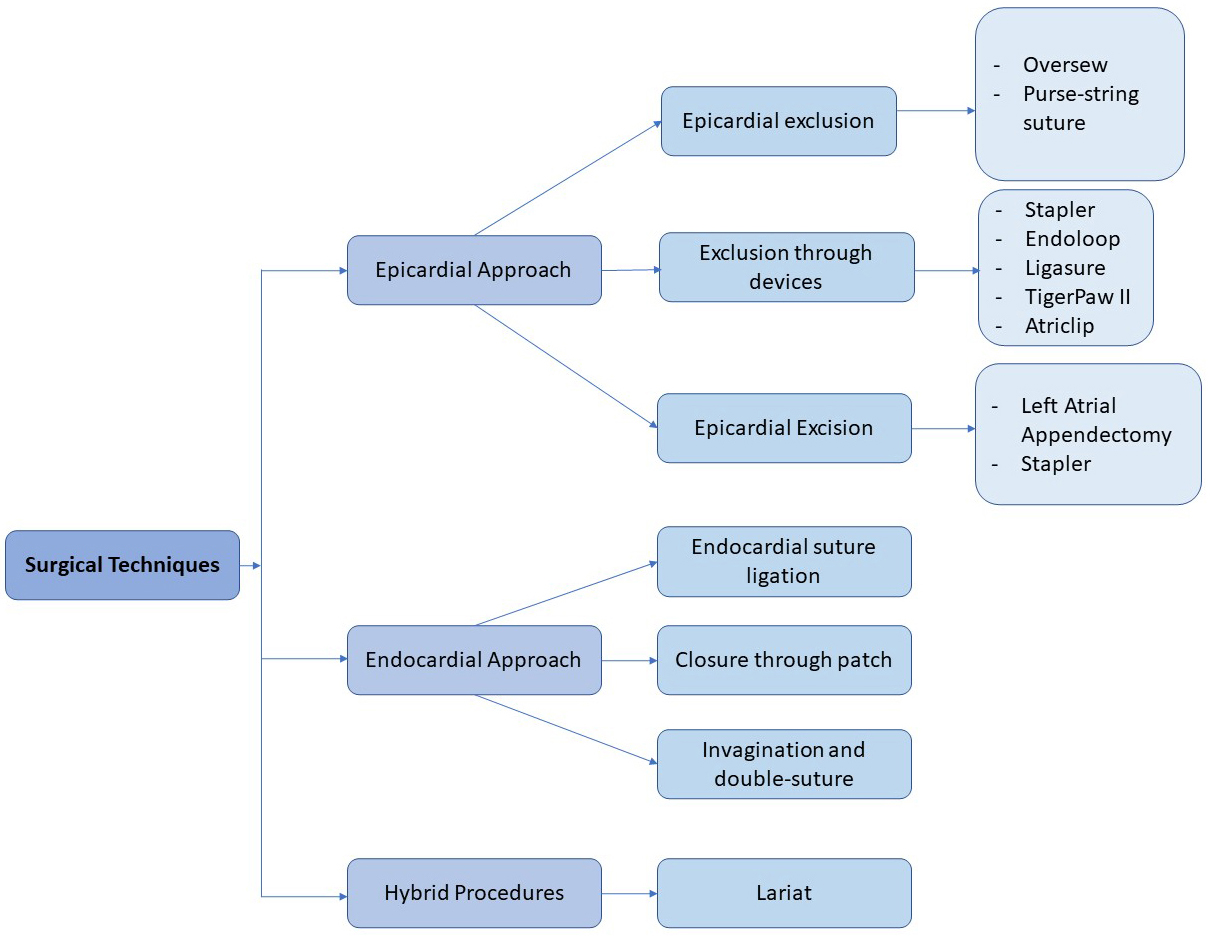
**Surgical left atrial appendage closure classification**.

**Fig. 3.**
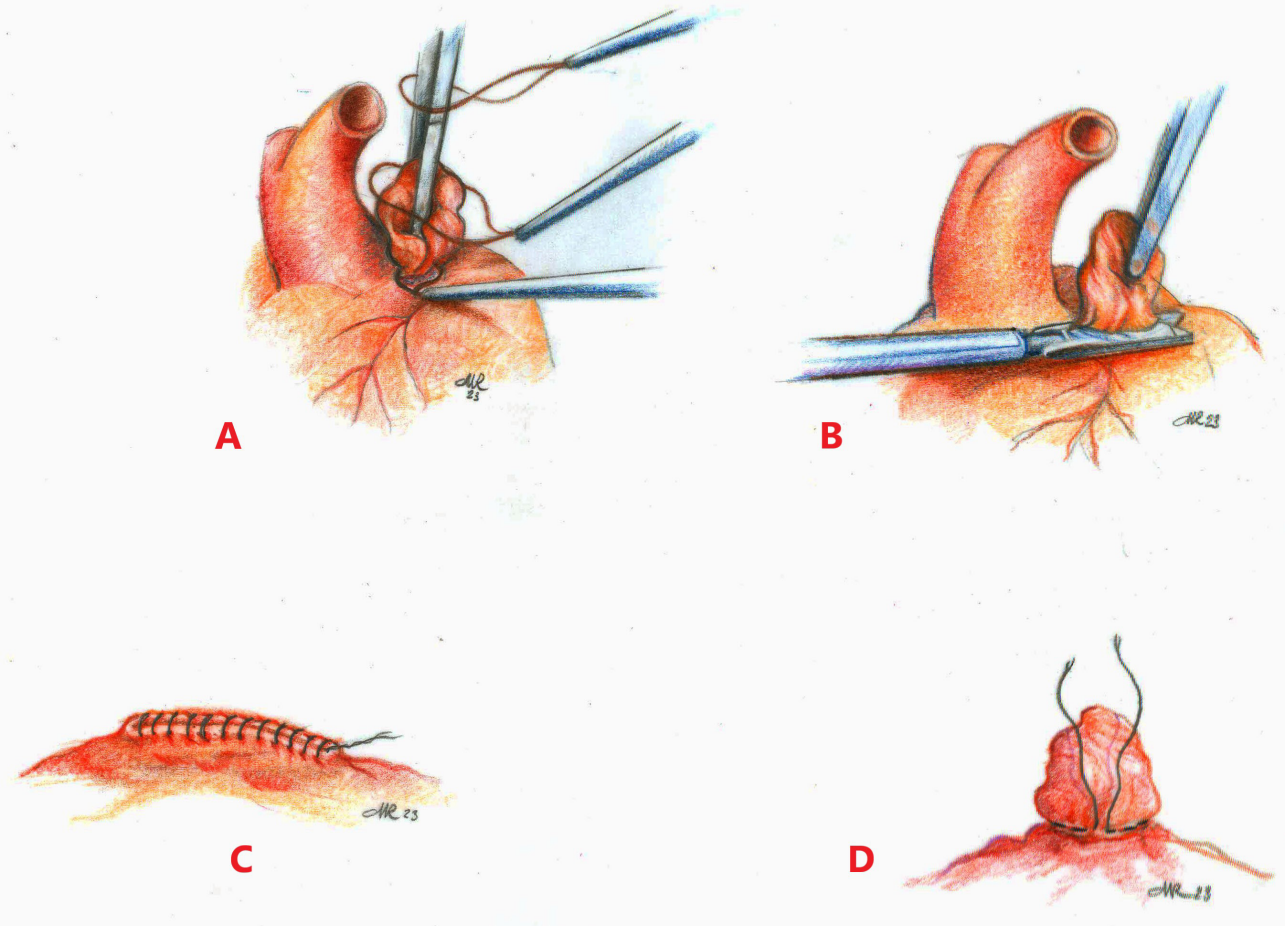
**Surgical techniques of left atrial appendage closure**. (A) Endoloop Snaring. (B) Surgical stapler. (C) Epicardial excision. (D) Purse string exclusion.

Different techniques have been described and adopted for the sLAAC such as epicardial exclusion (oversew, purse string, with or without polytetrafluoroethylene (PTFE) reinforcement), epicardial excision (with stapler, with stapler and excision of the left appendage, with or without reinforcement, with epicardial clips or through snares/suture loops), or endocardial suture ligation, with or without amputation [23] (Figs. 2,3). Another described technique, generally adopted in patients with a large base appendage, is the closure through autologous or bovine pericardial patches. The continuously rising interest in this procedure also led to the introduction of newer techniques, such as the invagination of the appendage in the left atrium, and hybrid techniques that combine a surgical and/or percutaneous approach to an endovascular one, such as the Lariat Device technique [24] (Fig. 2). Results of sLAAC are often confounding due to lack of standardized criteria for definition of Left Atrial Appendage Closure (LAAC) success. Different studies (see Table 1), in fact, tend to arbitrarily assess patency of the LAA with different criteria, that may be either more stringent (as in case of complete absence of flow and stump) or more permissive (Stump or Flow <1 cm). Therefore, the analysis of different techniques is particularly challenging, especially if we consider that only a few studies assessed the patency of LAA by comparing the different techniques. The aim of this systematic review is to evaluate the reported patency rates of different techniques, focusing on the possible bias associated with lower successful sLAAC, as well as to provide an introductive description of the different techniques to facilitate the evaluation and the outcomes analysis of the available surgical strategies.

**Table 1.** **Comparison of Surgical Left Atrial Appendage Closure Techniques**.

Author	Year	N. patients	N. patients at FU	Definition of complete closure	Time to mean echocardiographic FU	Intraoperative echocardiographic assessment	Intraoperative success rate	Follow-up assessment	Type of LAAC	Success rate
Kiankhooy MD *et al * [68]	2022	100	97	3	685	TEE	100%	TEE	Clip	96%
Shirasaka T *et al * [47]	2021	8	8	0	7	TEE	100%	TEE	Endocardial	100%
		6	6						Clip	100%
Fleerakkers J *et al * [39]	2020	13	13	0	-	TEE	100%	CT Scan	Clip	100%
Suematsu Y *et al * [33]	2020	43	43	0	90	TEE	100%	CT Scan	Clip	100%
Kats ES *et al * [61]	2000	50	20	3	1900	TEE	67%	TEE	Ligation	60%
Hirnle G *et al * [44]	2020	50	19	0	180	TEE	100%	TEE + CT Scan	Endocardial	95%
Tilz RR *et al * [57]	2020	138	103	2	181	TEE	94.2%	TEE	Lariat	97.10%
Lin B *et al * [40]	2020	74	74	0	200	-	-	TEE	Epicardial	72%
Güner A *et al * [67]	2020	101	101	3	90	-	-	TEE	Epicardial	65.3%
Ellis CR *et al * [46]	2020	33	33	0	30	TEE + Angiography	100%	TEE or CT Scan	Lariat	82%
Parikh V *et al * [35]	2019	108	80	0	365	TEE	93%	TEE	Lariat	96.3%
Mohanty S *et al * [45]	2019	306	306	0	30	-	-	TEE	Lariat	73.50%
Fu M *et al * [66]	2019	257	257	3	365	-	-	TEE	Stapler	76.70%
Caliskan E *et al * [64]	2018	291	23	3	1080	TEE	100%	CT Scan	Clip	100%
Van Laar C *et al * [31]	2018	222	222	0	180	TEE	-	TEE or CT Scan	Clip	95%
Park-Hansen J *et al * [30]	2018	101	10	0	524	-	-	TEE	Endocardial	100%
Fink T *et al * [59]	2018	44	35	3	183	TEE + Angiography	100%	TEE	Lariat	66%
Ohtsuka T *et al * [28]	2018	201	194	0	30	TEE	100%	CT Scan	Stapler	97.50%
Ellis CR *et al * [38]	2017	65	65	0	90	TEE	100%	CT Scan	Clip	93.90%
Kurfist V *et al * [63]	2017	101	Unknown	3	90	TEE	98%	TEE	Clip	100%
Cullen MW *et al * [29]	2016	93	93	0	30	TEE	-	TEE	Amputation	100%
									Epicardial	41%
									Endocardial	71%
									Stapler	71%
Lee R *et al * [60]	2016	28	21	3	140	TEE	87.5%–100%	TEE	Endocardial	57%
									Stapled Excision	100%
									Amputation	100%
Bartus K *et al * [53]	2016	58	48	1	90	TEE + Angiography	100%	TEE	Lariat	92.30%
Lakkireddy D *et al * [49]	2016	682	480	0	90	TEE	98%	TEE	Lariat	93.3%
Pillarisetti J *et al * [56]	2015	259	259	2	365	TEE	98%	TEE	Lariat	87%
Sievert H *et al * [34]	2015	139	127	0	45	TEE + Angiography	99%	TEE	Lariat	90%
Stone D *et al * [55]	2015	25	22	2	45	TEE + Angiography	100%	TEE	Lariat	100%
Aryana A *et al * [41]	2015	72	72	0	90	-	-	CT Scan	Ligation	64%
Emmert MY *et al * [37]	2014	40	32	0	1080	TEE	100%	CT Scan	Clip	100%
Miller MA *et al * [52]	2014	41	41	1	100	TEE + Angiography	93%	TEE or CT Scan	Lariat	76%
Price MJ *et al * [54]	2014	145	63	2	112	TEE	94%	TEE	Lariat	93%
Zapolanski A *et al * [62]	2013	808	56	3	-	TEE	100%	TEE	Ligation	94.70%
Bartus K *et al * [51]	2013	89	65	1	365	TEE	96%	TEE	Lariat	98%
Massumi A *et al * [50]	2013	20	17	1	96	TEE + Angiography	100%	TEE	Lariat	100%
Adams C *et al * [42]	2012	12	12	0	90	TEE	100%	CT Scan	Ligation	25%
Ailawadi G *et al * [36]	2011	70	61	0	90	TEE	95.7%	TEE or CT Scan	Clip	98.3%
Slater AD *et al * [58]	2011	60	54	3	90	TEE	93.3%	TEE	Tigerpaw II	100%
Salzberg SP *et al * [32]	2010	34	Unknown	0	90	TEE	100%	CT Scan	Clip	100%
Kanderian AS *et al * [69]	2008	137	137	3	243	-	-	TEE	Amputation	73%
									Endocardial	23%
									Stapler	0%
Healey JS *et al * [65]	2005	52	44	3	60	-	-	TEE	Stapler	72%
									Epicardial	45%
García-Fernández MA *et al * [43]	2003	58	58	0	2082	-	-	TEE	Ligation	89.70%
Johnson WD *et al * [3]	2000	437	Unknown					TEE	Stapler	100%
									Epicardial	100%

Definition of Complete Suture: (0) absence of leaks and of flow between LAA and left atrium; (1) residual flow ≤1 mm as complete LAAC; (2) residual flow ≤5 mm; (3) flow ≤10 mm or a residual stump <1 cm. LAA, left atrial appendage; FU, follow-up; TEE, transesophageal echocardiography; CT Scan, computed tomography scan; LAAC, left atrial appendage closure.

## 4. Methods

**Fig. 4.**
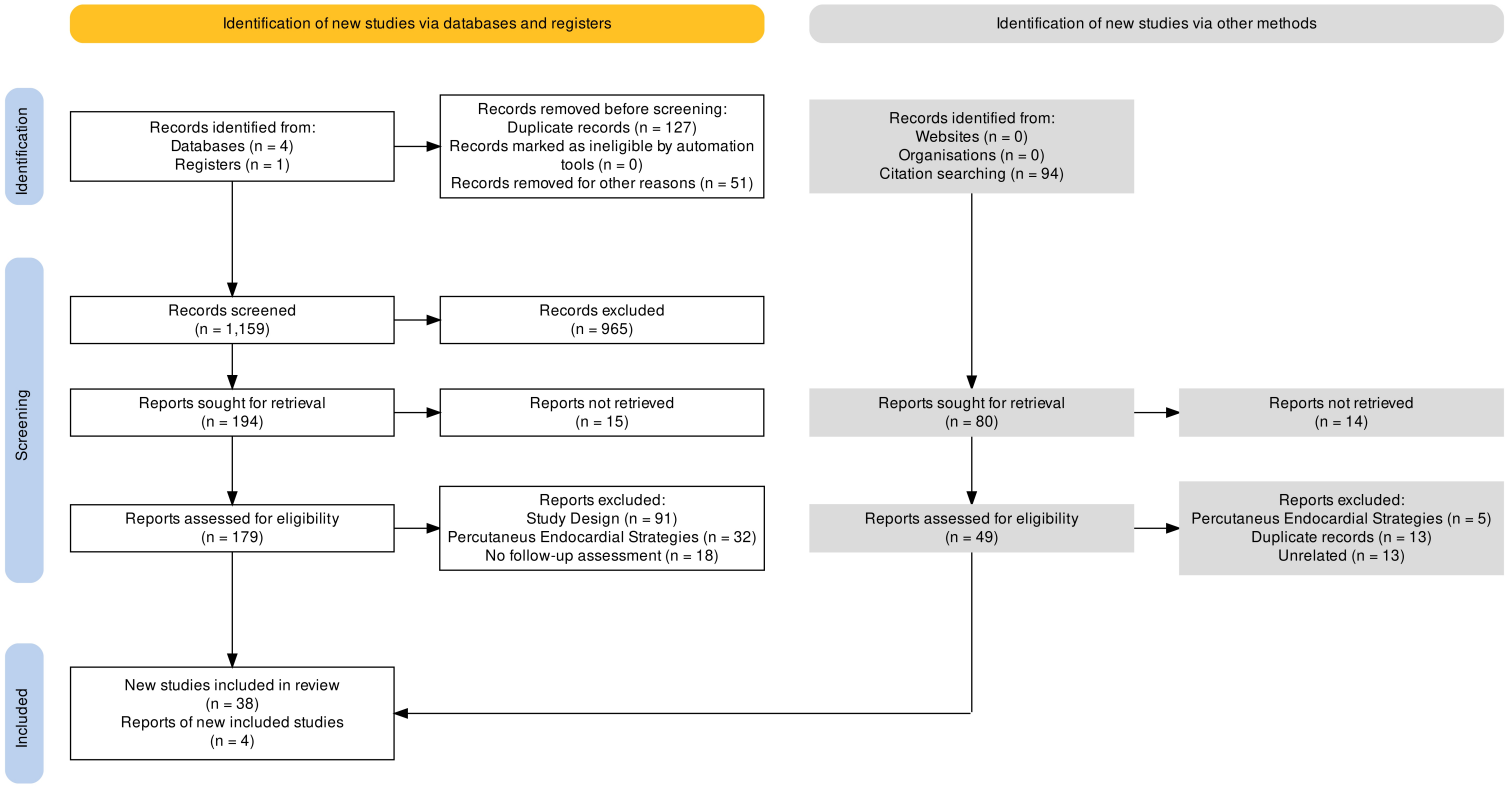
**PRISMA Chart**.

This review adhered to preferred reporting items for systematic reviews and meta-analyses guidelines (PRISMA) [25] and was performed in line with a prespecified protocol. Two independent investigators (MDA and SR) searched the PubMed, Scopus, Web of Science, Cochrane Central Register of Controlled Trials (CENTRAL), and OVID to identify relevant studies. The following key medical subject headings (MeSH) terms and Emtree terms were used: left atrial appendage, LAAC, surgical closure or surgical occlusion. The search was extended from inception up to December 31, 2022. Case reports, editorials, expert opinions, review articles, guidelines, animal studies and non-English studies were arbitrarily excluded (Fig. 4). Two investigators independently screened all titles and abstracts to identify studies that met the inclusion criteria and extracted relevant data. After this primary evaluation, two authors personally screened the reference list of previous reviews and metanalysis to identify possible eligible trials. Consecutively, a PICO (Population, Intervention, Comparison and Outcomes) strategy assessment of literature [26] was performed to search eventual other relevant studies that may have been ignored. The following terms were used for analysis: P (atrial fibrillation); I (surgical left atrium appendage closure); C (left atrium appendage closure); O (complete closure) (Fig. 4). Once individual studies were identified, efficacy and safety data were represented in the form of a simple pooled analysis. The lack of control groups in individual studies limited our ability to perform a meta-analysis of the presented data. Therefore, statistical significance for each measured variable could not be generated. Microsoft Excel (Microsoft 365 MSO, version 2305 Build 16.0.16501.20074, Microsoft Corporation, Redmond, WA, USA) was used for all data analysis, with categorical data expressed as frequencies and percentages (%) and continuous data expressed as mean. The risk of bias of this analysis was assessed by using the ROBVIS (Risk-of-bias VISualization) [27].

## 5. Results 

Two independent investigators (MDA and SR) extracted the following data from the included studies: authors, year of publication and baseline features, including type of surgical LAAC, time to follow-up evaluation, type of echocardiographic assessment, whether intraoperative assessment was performed and eventual imaging modality, rate of LAAC success. Since definition of LAAC success was not standardized and the results in terms of outcome differ between authors, the criteria used to determine LAAC success was included in the dataset. Literature search retrieved 1337 + 94 relevant reports, of which 42 included analyses of complete LAAC (cLAAC) at follow-up after surgical closure (Fig. 4).

### 5.1 Patients Baseline Characteristics

Forty-two studies (see Table 1) were included in our analysis. The total number of patients underwent sLAAC was 5671, and in 3471 (61.2%) an imaging follow up was performed. Mean imaging follow-up was performed at 299.8 days (7 to 2082 days). Transesophageal echocardiographic (TEE) evaluation was performed in 28 of 42 studies, cardiac computed tomography scan (CT scan) in 10 of 42 studies, while 4 studies combined both imaging strategies according to patient characteristics, namely chronic renal impairment and stumps at TEE assessment (see Table 1).

However, these studies differ in patient selection methods, design, and, most importantly, definition of success rate.

### 5.2 Procedure Success

Different definitions of success rate have been adopted by different authors for evaluation of complete LAAC closure (cLAAC) at follow-up assessment. From 42 studies on this topic, 21 defined complete closure as full absence of leaks and flow between LAA and left atrium [28, 29, 30, 31, 32, 33, 34, 35, 36, 37, 38, 39, 40, 41, 42, 43, 44, 45, 46, 47, 48, 49]; 4 studies suggested a residual flow ≤1 mm as complete LAAC [50, 51, 52, 53]; 4 studies considered acceptable a residual flow ≤5 mm [54, 55, 56, 57], while 12 studies were more permissive and considered a cLAAC when flow was ≤10 mm or a residual stump <1 cm was detected [58, 59, 60, 61, 62, 63, 64, 65, 66, 67, 68, 69] (Table 1, Ref. [3, 28, 29, 30, 31, 32, 33, 34, 35, 36, 37, 38, 39, 40, 41, 42, 43, 44, 45, 46, 47, 49, 50, 51, 52, 53, 54, 55, 56, 57, 58, 59, 60, 61, 62, 63, 64, 65, 66, 67, 68, 69]). Data were not available for one study [3].

Of all valuated studies, 6 compared results of different techniques, while 36/42 reported success rate of the procedure. Clip deployment was valuated in 11 studies [31, 32, 33, 36, 37, 38, 39, 47, 63, 64, 68], reporting a mean success rate of 98% (range 93.9%–100%). Intraoperative imaging assessment of effectiveness of cLAAC was observed in 100% of patients (range 95.7%–100%), with a total population of 985 patients, of which 562 were also evaluated at follow-up. Fourteen studies [34, 35, 45, 46, 49, 50, 51, 52, 53, 54, 55, 56, 57, 59] investigated Lariat procedure (SentreHEART, Inc., currently Atricure Inc. Redwood City, CA, United States) and reported mean follow up rates of 88% (range 66%–100%), while intraoperative success was obtained in 98% of patients (range 93%–100%). A total of 2087 patients underwent Lariat procedures, and 80% underwent imaging follow-up. Surgical amputation was evaluated in 3 studies [29, 60, 69], with a mean success rate of 91% (range 73%–100%). Complete LAA closure through endocardial suture was obtained in 74.3% of cases (range 23%–100%), analyzed in 6 studies [29, 30, 44, 47, 60, 69], while Epicardial suture [3, 29, 40, 65, 67] obtained a success rate of 65% (range 41%–100%). LAAC ligation, evaluated in 5 studies [41, 42, 43, 61, 62], has proven to be a little more efficient, with a success rate of 60.9% (range 25%–94.7%). Results of the stapler technique were analyzed both with and without excision of the LAA: stapler exclusion with excision of the LAA was evaluated only in 1 study [60] with a success rate of 100%, while stapler without excision, evaluated in 6 studies [3, 28, 29, 65, 66, 69], had a success rate of 70%, although it may be considered less predictable (range 0%–100%).

## 6. Techniques Description

The techniques of sLAAC can be divided according to the surgical approach into epicardial, endocardial and hybrid procedures (Figs. 2,3).

Within the macro-area of epicardial surgical approach to LAAC, we may identify exclusion techniques, with sutures or devices and excision techniques. Oversew is one of the simplest techniques to perform LAAC: it’s based on the mobilization of the base of the LAA, to increase the distance between the base of the suture and the circumflex artery, followed by the closure of the LAA with a vascular clamp, therefore 2 nonabsorbable, braided, 2-0 ligatures are applied and knotted with a distance of 5 mm from each other [70]. Another possible epicardial suture technique is the Purse-string suture exclusion (Fig. 3D): the base of the LAA is carefully mobilized and a purse string suture (generally nonabsorbable, braided, 2-0 suture), mostly reinforced with PTFE felt pledgets, is placed and then tied at the base of the LAA [71].

Different devices have been used and validated to LAAC. Surgical non-cutting staplers, with or without pericardial buttressing, require careful positioning at the neck of the LAA, with the closing mechanism that provides a rapid and precise closure of the appendage. A bovine pericardial strip can be used to reinforce the staple line. Endoloop snaring consists in a detachable snare loop (Endoloop) positioned at the base of LAA and secured [72] (Fig. 3A). LigaSure vessel-sealing system (epicardial welding) (LigaSure ${\mathrm{Xtd}}^{T\,M}$, Valleylab, Louisville, KY, USA) uses radiofrequency energy through a bipolar device to the LAA base, thus ensuring tissue fusion and scarring, leading to the obliteration of the LAA [73]. TigerPaw II system (interrupted, mattress suture-based epicardial fastening) (Maquet, Inc., Rastatt, Germany) was recalled from the U.S. Food and Drug Administration (FDA) on May 7, 2015. It was an implantable soft silicone occlusion fastener positioned epicardially around the base of the LAA, that produced interrupted, mattress sutures [58]. AtriClip LAA exclusion system (epicardial clipping) (AtriCure, Inc., West Chester, OH, USA) is used for surgical closure of the LAA under direct vision, and is a repositionable clip preloaded on a single-use device. The peculiar knit braided polyester is supposed to prevent cutting/damaging of the LAA, therefore reducing complications. It is provided in 4 different sizes (35, 40, 45 and 50 mm) [32].

Epicardial excision techniques include left atrial appendectomy/surgical cut-and-sew amputation, a strategy based on the amputation of the LAA at the base by excision, and the neck of the LAA is therefore oversewed in multiple fashions, such as running/mattress suture, with single or double layers, with or without pledgets reinforcement (Fig. 3C). Surgical cutting staplers, with or without pericardial buttressing, which is the same procedure described for non-cutting stapler, but in this case the appendage is removed (Fig. 3B).

Different endocavitary techniques have been described to perform LAAC. One of the most used is Endocardial suture ligation, with or without amputation: through a left atriotomy, a single or double-layer suture is placed at the base of the LAA, in a running or mattress-like fashion. Another possibility is an Endocardial Purse-String Suture in which an endocardial suture ligation is generally performed with a monofilament, non-absorbable 3-0 suture, with or without PTFE reinforcement [74]. Other described techniques are Closure through autologous or bovine pericardial patch, performed in cases of a large base of the LAA: a patch exclusion may be performed through a non-treated autologous or bovine pericardium patch using 4-0 polypropylene running suture technique; and Invagination and double-suture technique, performed through invagination of the LAA, generally though suction [48], into the LA, with 2 purse-string sutures positioned at the base to permanently prevent its evagination [75].

Hybrid procedures combine a percutaneous, generally subxiphoid, approach to an endocardial one and in this regard Lariat procedure (SentreHEART Inc, Redwood, CA, USA) is the most performed. Access to the pericardium is performed through a subxiphoid approach, with needle directed in anterior-lateral direction. An occlusion balloon, back loaded with a magnet-tipped endocardial guide wire, is positioned in the LAA through transseptal puncture under fluoroscopic guidance. The magnet-tipped epicardial guide wire is inserted into the pericardial space and attached to an endocardial magnet-tipped guide wire. The snare is then advanced over the epicardial wire and positioned over the LAA. Snare positioning at the ostium of the LAA is guided by balloon location at the opening of the LAA and confirmed with TEE assessment. The snare is therefore closed and a left atriogram is performed to assure the absence of a stump. Surgical suture is tightened to ligate and exclude the LAA. The Lariat snare is removed from the pericardial space and cutted [53]. Evidence on the results of sLAAC is mostly derived from non-randomized case series, monocentric observational cohort studies or retrospective registries with limited follow-up. Those results are often marginal and sometimes conflicting, and there is a selection bias due to the limited population of this studies compared to the wide use of LAAC devices. However, current guidelines for the management of AF still recommend surgical occlusion or exclusion of the LAA for stroke prevention in patients with contraindication for long-term anticoagulant treatment (Class IIb, Level B) and in patients undergoing cardiac surgery or thoracoscopic AF surgery (Class IIb, Level C) [76].

## 7. Discussion

To identify eventual biases and highlight possible failures of the described techniques, here we present a discussion of the studies with lower success rates.

Cullen and colleagues [29] retrospectively reviewed patients undergoing TEE within 30 days from cardiac surgery and surgical LAA to guide cardioversion. Their rate of LAA patency was higher after suture closure compared to surgical excision or stapler closure, with an overall incidence of patency of 37%. One of the possible biases in this study is the small number of patients for the different surgical techniques (7 patients underwent Stapler exclusion, 23 LAA amputation), associated with the retrospective nature of this study, and the selection of patients who experienced post-operative AF.

Kanderian and colleagues [69] studied 137 of 2546 patients who underwent LAA closure with TEE follow-up. An exclusion technique was adopted in 52 (38%), while 85 (62%) received an excision procedure, of which 80% had scissor excision and oversew and 20% had cutting stapler excision. Rate of successful closure reached 40%, with 60% of suture exclusions failed due to persistent flow on TEE, and 60% of the stapled exclusion failed for large remnant. This study indeed highlighted the importance of a complete LAA occlusion, since 41% of the patients with unsuccessful closure had LAA thrombus formation compared with 0% of the successful closure group and 0% of the excision group. This well-known study reports the lowest success rate in literature, especially regarding exclusion endocardial suture, that has a success rate of 23%. Possible biases of this studies regard patient selection and the retrospective nature of the study: only patients that underwent TEE for other causes (that include, aortic dissection, transient ischemic attack [TIA], endocarditis, left ventricular thrombus) were included, therefore only 5.4% of patients that underwent surgical LAAC were evaluated. Suboptimal patency rate for internal ligation was evidenced also by Lee and colleagues [60]: even if it was performed on a small number of patients (N = 8), it evidenced a patency rate of 43%. Other techniques valuated in this study designed as a randomized, prospective trial (Amputation and Stampler Excision), revealed good outcomes with a complete closure of 100%.

Katz and colleagues [61] studied 50 patients who underwent LAA ligation and concomitant mitral valve surgery. The technique applied for LAA ligation was the endocardial suture exclusion, and the results evidenced that 36% of LAA ligations were incomplete: 50% of the unsuccessful closures had spontaneous echo contrast or thrombus in the LAA and 22% had thromboembolic events.

García-Fernández and colleagues [43] reviewed 58 patients that underwent mitral valve surgery and concomitant LAA ligation, with a group control of 147 patients who underwent isolate mitral valve surgery. The incompletely occluded LAAs rate was 10.3%, with evidence that no LAA occlusion and incomplete LAA occlusion were major risk factors for the development of thromboembolic sequelae at follow-up.

Among studies valuating Lariat procedure, 2 evidenced significantly worst success rates [52, 59]. Fink and colleagues [59] performed a retrospective study in 44 patients that underwent LAA ligation with Lariat, with 35 patients that underwent TEE follow-up, and a patency rate of 34%. As stated by the authors, the institution had no previous experience with this device, and this may explain the obtained results, since those patients may be part of the learning curve of the center. Miller and colleagues [52] experienced similar patency rates (74%). Their analysis, even if performed on a small population (41 consecutive patients), included 4 centers, therefore few procedures were performed at each center: possible learning curve may be evidenced also by the high rate of complication, in particular perforation of LAA, that occurred in 9% of the patients, with 50% of them requiring open surgical correction.

Two studies reported low success rate for epicardial surgical ligation [41, 42]. Adams and colleagues [42] performed LAA ligation with an Endoloop® suture ligature (Johnson & Johnson, Cincinnati, OH, USA) on 12 patients. Surgery was performed by a single operator, and, at 3-month follow-up, CT scan evidenced a rate of LAA patency of 75%, even if intraoperative TEE was negative. As correctly stated by the authors, possible explanation for these findings may be: edema of the LAA, induced by cardiopulmonary bypass, that reduces over time, possibly leading to a re-establishment of a connection between LA and LAA; suture ligature not placed deep enough on the base of the LAA due to concern to the circumflex coronary artery. One possible bias may be in the choice of CT for follow-up evaluation, that, as stated, maybe too sensitive in detecting a communication, and no TEE evaluation was performed to complete the analysis. Aryana and colleagues [41] valuated, with CT angiography imaging, 72 patients after LAA ligation in conjunction with mitral valve/AF surgery in a single-center, nonrandomized analysis. Surgical ligation was performed by 5 experienced operators with an oversewing technique with a double-layer of running Prolene suture. As stated from the authors, CT angiography has not been validated as the test of choice for LAAC closure assessment; however, it was able to detect incomplete LAAC in 24% of the patients, with a residual stump in 12% of the patients. Oversewing technique evidenced not-so-brilliant results also in the analysis of Lin and colleagues [40] and Güner and colleagues [67]. The former was a retrospective analysis of 193 patients that underwent TEE after surgical LAAC for any reason. The oversewn technique was performed with a double layer of running suture with or without excision of the LAA (and without any reference of relative frequencies). The main bias of this study, as correctly stated from the authors, is that of patient selection, since only patients that required TEE for any reason were included in this study, including possible endocarditis and stroke/TIA (8/74) [40]. Similar results were evidenced also by Güner and colleagues [68], with a procedural success rate of 65.3%. This multicentric, retrospective study, analyzed oversewing technique with a double-layer of running prolene suture. The inability to review TEE of all patients that underwent LAAC limited the population to 101 patients, therefore the percentages might not be representable to all patients undergoing surgical LAAC.

Stapler devices were valuated both in the studies of Fu and colleagues [66] and Healey and colleagues [65].

The study performed by Fu and colleagues [66] is a single-center, prospective cohort study that assesses the safety and efficacy of thoracoscopic LAA. LAAC was performed on 257 consecutive patients with a thoracoscopic-assisted bilateral intercostal approach, without cardiopulmonary bypass. The stapler used (Johnson & Johnson EZ-45G, New Brunswick, NJ, USA) employs 2 lines of staples to resect and suture the LAA. At 3- and 12-months TEE assessment, success rate was 76.7%, considered as eventual residual stump <1 cm. This study demonstrates the efficacy of LAAC closure compared to warfarin for stroke prevention, but it does not investigate the low success rate of a stand-alone procedure. The LAA Occlusion Study (LAAOS), designed by Healey and colleagues [65], randomized 77 patients in a control group (N = 25) and an occlusion group (N = 52), performed by epicardial suture or stapler, with TEE follow-up. The rate of closure success was of 43% in the suture group and 72% in the stapled group, with evidence of failure in the epicardial suture group due to inadequate technical closure, while in the stapled group for residual remnant size. In this study, overall perioperative stroke rate was 2.6% (2 of 77), suggesting possible benefit of LAA occlusion.

Overall, surgical rates of complete closure in our analysis were 82%, with variable results depending on the surgical technique used. In addition, most of the studies included in this analysis are retrospective in design and performed mostly in single centers, and tendentially with few patients, therefore wariness must be practiced about sLAAC failure rates.

Some explanations have been proposed for the high rate of incomplete sLAAC, and in particular for endocardial suture: cautious suture bites may be positioned a little higher and more superficial in the atrial wall to avoid the circumflex artery and there may be a technical difficulty to reach the distal edge of the LAA, in particular in patients that present a mitral valve annuloplasty ring or prosthesis [77]. Furthermore, oedema due to the surgical gesture may justify LAA recanalization at follow up, and different LAA morphologies may be responsible for an incomplete closure of the ostium. Internal ligation is mostly associated with gap at follow-up, due to tears through the tissue, especially if the patient is in sinus rhythm, while excision, either surgical or stapled, are mostly associated with stump evident at intraoperative evaluation [60].

Difficulty in evaluating sLAAC may be due also to the different thresholds used to identify incomplete closure by different authors, and to the absence of a routinary TEE evaluation at follow-up. The recently published SCAI/HRS Expert Consensus Statement on Transcatheter LAAC [78] identifies >5 mm a critical threshold for peri-device leaks (PDLs), therefore considered significant, with recommendation to continue oral anticoagulant therapy, with an incidence of PDLs between 11 and 57%, depending on the implanted device and the imaging modality used [79]. Those cut-offs are therefore well described, with literature evidence to support the thresholds, and universally accepted for endocardial LAAC: similar results are not described for sLAAC, increasing the variability in the evaluation of this procedure. Those guidelines also recommend TEE or cardiac computed tomography at 45 to 90 days after LAAC to assess for peri-device leak and device-related thrombus, but similar directives have not yet been proposed for surgical closure, nor have been published recommendations from the cardiothoracic society to guide surgical treatment of LAA.

Evidence of the potential benefits of sLAAC are results of LAAOS III (Left Atrial Appendage Occlusion Study) [80], which is a large prospective, multicenter Randomized Controlled Trial (RCT) evaluating the effect of LAAC on neurological complications. This study evidenced that, in AF patients that underwent cardiac surgery, sLAAC was associated with lower incidence of neurologic complications. This study, however, did not perform an imaging evaluation of LAAC closure at follow-up, therefore it adds little to the evaluation of the different surgical techniques. Furthermore, a magnitude of techniques were accepted, and no evaluation is performed based on the surgical strategy adopted.

The same issue, the absence of LAAC evaluation at follow-up, may be evidenced in another recent, prospective, multicenter RCT, the ATLAS Study (AtriClip® Left Atrial Appendage Exclusion Concomitant to Structural Heart Procedures) [81] that evaluates the impact of post-operative AF in patients that had no surgical LAAC and patients who underwent LAAC with AtriClip. This study recruited patients with no previous history of AF, although this “protective” treatment is not recommended by latest guidelines. ATLAS demonstrated the safety and effectiveness of Atriclip for LAAC and a potential protective effect of LAAC on postoperative AF (POAF): in LAAC group, even if POAF rate was higher, incidence of thromboembolic rate was lower.

Hopefully, a definitive answer on the role of preventive LAAC in patients undergoing cardiac surgery for another indication will be provided by the Left Atrial Appendage Exclusion for Prophylactic Stroke Reduction (LeAAPS) (NCT05478304) [82] that will evaluate thromboembolic events in 6500 patients with increased risk for stroke and AF, randomly assigned to LAAC with AtriClip or not. Evaluation at follow-up of results of surgical closure, however, will not be included in the primary outcomes [83].

## 8. Study Limitation

**Fig. 5.**
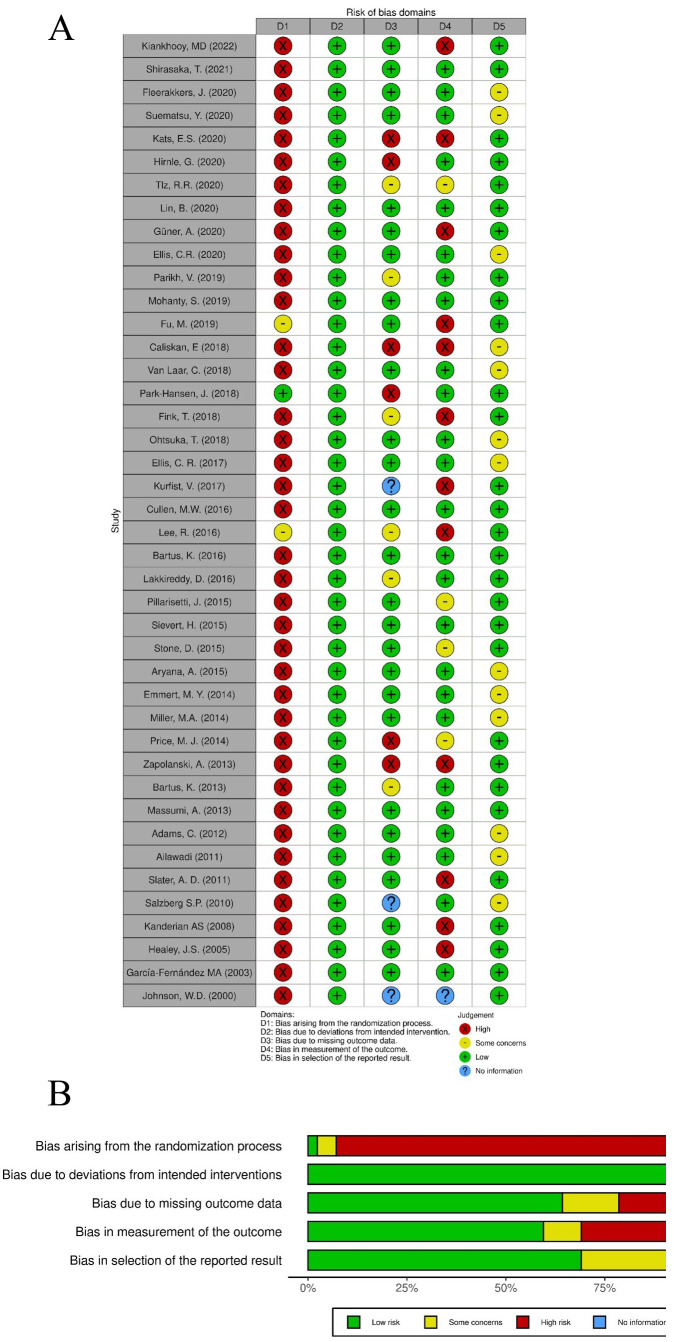
**ROBVIS: Risk-of-bias VISualization**. (A) Traffic Light Plot for risk of bias domains. (B) Weighted bar plots of the distribution of risk-of-bias for each domain.

Several biases may be evidenced in this review, as summarized in Fig. 5 (Fig. 5A,B). In particular, the studies reported are mostly single center, retrospective case series, where no randomization is performed. Therefore, to estimate the risk of bias of this analysis, we used the ROBVIS (Risk-of-bias VISualization) [27].

As expected, considering the typology of article included in this review, a selection bias is particularly evident: not all patients included in the studies were included in the analysis, contrarily to what is expected from a target randomized trial. Considering the post-intervention domains, the most urgent bias may be a bias due to missing data, since follow-up evaluation is not complete for all individuals initially included and followed, and particularly a bias in measurement of outcomes, since the reported cut-off for LAAC failure differ greatly from different authors, and also the modality of imaging varies in the different studies. In addition, most of the studies reported evaluate LAAC combined with other surgical procedures, and, as stated from the Cochrane Risk of Bias guidance [84], co-interventions are a potentially important source of bias, even if stand-alone LAAC has been validated only in the most recent guidelines [76].

In general, this review highlights the absence of unified criteria for LAAC complete closure, that does not allow a proper comparison of the results described in the scientific literature with different techniques, along with absence of LAAC evaluation as primary endpoint in most of the reported studies: patients frequently underwent imaging follow-up due to clinical reason, and therefore this may alter the reported results of the surgical procedure.

## 9. Conclusions

The increasing prevalence of AF and the increased morbidity and mortality related to thromboembolic stroke have resulted in intensive research on stroke prevention and stroke related-risk reduction strategies, with a renewed interest in the possible surgical strategies for LAA occlusion. These techniques, initially performed only as a concomitant procedure during open-heart surgery, are now including some stand-alone surgical procedures in minimally invasive settings to directly address LAA.

However, data on the safety and feasibility of surgical LAA occlusion are poor and with conflicting results.

Evaluation of surgical techniques, their standardization, univocal cut-offs for failure and a definite follow-up assessment are essential to increase the reproducibility and therefore expand the potential of this procedure. If further validated on large-scale non-retrospective and multicentric studies, this promising surgery may offer a valuable alternative for patients with AF and ineligible for oral anticoagulation therapy.

## Supplementary Material

Supplementary material associated with this article can be found, in the online version, at https://doi.org/10.31083/j.rcm2406184.
